# Supplementary material for: Sociodemographic predictors of health-related quality of life dimensions in hypertensive patients: A cross-sectional study in a Ghanaian Municipal Hospital
Source: PLOS Glob Public Health. 2026 Mar 17;6(3):e0006140. doi: 10.1371/journal.pgph.0006140 (PMC12994814; doi:10.1371/journal.pgph.0006140)
Supplement: Attachment — Submitted filename: Response_to_Reviewers_PGPH-D-25-04048.docx [file pgph.0006140.s002.docx]

**Response to Reviewers – Manuscript ID: PGPH-D-25-04048**

**Title:** Sociodemographic predictors of health-related quality of life dimensions in hypertensive patients: A cross-sectional study in a Ghanaian Municipal Hospital

**Journal:** PLOS Global Public Health

We would like to sincerely thank Dr. Theingi Maung Maung (Academic Editor) and the reviewers for their time, effort, and constructive feedback on our manuscript. We have carefully considered all comments and have revised the manuscript accordingly. Below, we provide a detailed, point-by-point response to each comment. Changes made in the revised manuscript are highlighted using track changes, and the line numbers/pages where changes were made are indicated.

**General Acknowledgement**

We greatly appreciate the reviewers’ careful evaluation of our work. Their comments helped improve the clarity, rigor, and overall quality of the manuscript. In particular, we have:

1. Clarified the study population and sampling method.
2. Addressed all points regarding methodology, including hypertension definition, comorbidities, and data collection procedures.
3. Revised wording throughout to replace causal terms such as “influence” or “predictor” with neutral terms such as “association.”
4. Expanded the limitations section to acknowledge the potential selection bias and the lack of non-hypertensive controls.
5. Standardized all income categories and table captions for consistency and clarity.
6. Ensured that all cited references are accurate, peer-reviewed, and current.

**Point-by-Point Response to Reviewers’ Comments**

**Reviewer #1**

**Comment 1:** Please clarify how hypertension was defined and confirmed in this study.
**Response:** We have added a clear definition of hypertension and explained the confirmation process based on clinical records and WHO criteria.
**Line/Page:** [Lines 125-127; page 5]

**Comment 2:** Please clarify whether patients with common comorbidities were included, as these may affect QoL.
**Response:** We have specified inclusion/exclusion criteria regarding comorbidities and noted their potential confounding effect in the limitations.
**Line/Page:** [lines 132-136; page 6]

**Comment 3:** Explain how the total population (N = 1,280) was identified.
**Response:** Detailed description of population identification from outpatient registry, inclusion period, and follow-up criteria has been added.
**Line/Page:** [lines 141-144; page 6]

**Comment 4:** Describe the simple random sampling process in detail.
**Response:** Methodology section now includes the sampling frame, randomization procedure, and handling of duplicates or repeated visits.
**Line/Page:** [lines 150-155; page 6]

**Comment 5:** Clarify interviewer training and supervision, and standardization of data collection.
**Response:** Added details on interviewer training, supervision, and standardization procedures to ensure data consistency.
**Line/Page:** [Lines 191-197; page 8]

**Comment 6:** Explain whether WHOQOL-BREF was translated or culturally adapted.
**Response:** We added details on translation and cultural adaptation procedures, including back-translation and pretesting.
**Line/Page:** [Lines 181-184; pages 7-8]

**Comment 7:** Revise Results to highlight main findings from each table rather than repeating all numbers.
**Response:** Results section has been revised for clarity, emphasizing key findings.
**Line/Page:** [Lines 230-234, 237-243, 248-255; pages 9-14]

**Comment 8:** Avoid causal wording; use “associated factors” instead of “predictors.”
**Response:** All causal terms in the manuscript have been replaced with neutral language reflecting associations.
**Line/Page:** [Lines 89-92, 265-271, 279-280, 288-295, 305-306, 310-315, 319-320, 328-354, 369-370, 395-411 ; pages 4, 23-28]

**Reviewer #2**

**Comment 1:** Expand the limitations regarding recruitment from outpatient registry and the lack of non-hypertensive controls.
**Response:** Limitations section now explicitly discusses potential selection bias and the inability to isolate hypertension effects from sociodemographic confounders.
**Line/Page:** [Lines 382-388; pages 27]

**Comment 2:** Standardize income categories across text and tables.
**Response:** Income categories have been made consistent across the manuscript.
**Pages/Results:** [10-22]

**Comment 3:** Revise causal phrases and verify citations for 2025 references.
**Response:** All causal phrases were replaced with neutral language; DOIs and citations have been checked and corrected.
**Line/Page:** [Lines 89-92, 265-271, 279-280, 288-295, 305-306, 310-315, 319-320, 328-354, 369-370, 395-411 ; pages 4, 23-28]

**Comment 4:** Minor grammar, clarity, and abbreviation corrections.
**Response:** All minor language, grammatical, and abbreviation inconsistencies have been corrected. (**General acknowledgement)**

**Comment 5:** Explain rationale for median cutoff (≥50 = good QoL).
**Response:** Added explanation for the median cutoff selection based on previous literature.
**Line/Page:** [Lines 213-217; page 9]

**Comment 6:** Define abbreviations in table captions (e.g., KW for Kruskal-Wallis).
**Response:** All table captions now include definitions for abbreviations.
**Line/Page:** [Lines 244-245; page 12]

**Additional Notes**

**Data Availability:** All relevant data supporting the findings of this study are publicly available in the Zenodo repository at: <https://doi.org/10.5281/zenodo.18720533>

**Financial Disclosure:** Updated: “The authors received no specific funding for this work.”

We sincerely thank the editor and reviewers again for their thoughtful comments, which have strengthened our manuscript. We hope that these revisions satisfactorily address all concerns and that our manuscript is now suitable for publication in PLOS Global Public Health.

**Godfred Darko*: Corresponding and Lead author**

**darkogodfred10@gmail.com**
